# Artificial intelligence in breast pathology – dawn of a new era

**DOI:** 10.1038/s41523-023-00507-4

**Published:** 2023-01-31

**Authors:** Sunil S. Badve

**Affiliations:** 1 Department of Pathology and Laboratory Medicine, Emory University School of Medicine, Atlanta, GA USA; 2 Emory University Winship Cancer Institute, Atlanta, GA USA

**Keywords:** Breast cancer, Breast cancer

## Abstract

Artificial intelligence methods are been increasingly used for analysis of pathology slides. In this issue of the Journal, Sandbank et al. describe the validation and utility of a robust second reader system that can distinguish in situ and standfirst invasive carcinomas from non-neoplastic lesions of the breast.

The field of pathology is challenging with discordance noted even amongst expert pathologists. Although subjectivity and discordance amongst experts are inherent in the field of medicine, discordance amongst pathologists have been traditionally viewed as a matter of grave concern. This is in part due to the fact that pathologic analysis forms the foundation for disease management. A diagnosis of cancer or a benign proliferation or presence or absence of predictive biomarker may result in a dramatic change in therapeutic options as compared to regimen A or B, particularly when there is equipoise. Thus, the need for objectivity in pathologic analysis has been clearly voiced by oncologists and pathologists alike.

The search for objectivity has led to the development and popularity of gene expression signatures, which although in some cases are no better than histologic grade, and provide objective numerical values for risk of recurrence. This subjectivity of grade was further highlighted to promote the objectivity of molecular assays. This has recently come back full circle with the adoption of multi-parametric scores such as RSClin, which calls for the incorporation of two subjective parameters (tumor size and grade) with the 21-gene recurrence score in prognostic determination^1^. It goes without saying that greater objectivity will promote better prognostication.

Artificial intelligence (AI) in pathology (also called Pathomics) has blossomed in to a strong discipline wherein objectivity can be achieved. Whole slide images (WSI) can be generated with relative ease and made available to data scientists, who can extract 1000 s of features from these images. These features are correlated with biologic phenotype to create algorithms that enable recognition of phenotype, akin to that in genomics. In the early days, a variety of machine learning methods such as support vectors, and random-forests, were deployed, however, convoluted neural networks (CNN) has become the workhorse of pathomic analysis. CNNs are designed to use multi-level image structure, where basic image features such as contours are defined by changes in neighborhood pixel intensities and larger patterns are effectively successive combinations of smaller ones^2^. CNNs make predictions directly from images without relying on manually engineered intermediate steps; the image is gradually transformed in to a set of features that can be used for algorithm development. CNN-based algorithms have been successfully used for tumor detection, classification and prognostication as well as predicting response to therapy^2–4^.

Although theoretically simple, the AI-based analyses are complicated by the fact that these algorithms detect everything on the slides including scratches, ink-dots, dust marks and fingerprints. The analysis is also dependent on a number of pre-analytic and analytic factors including section thickness, the tinctorial characteristics of the H&E (hematoxylin and eosin) stain, and scanners used. Therefore, although the literature is full of examples of successful algorithms for tumor classification and prognostication, many tend to do poorly when applied to external cohorts. This “domain shift” needs to be mitigated before an algorithm can be clinically successful.

As of June 2022, a wide range of Artificial Intelligence (AI) as a Medical Device (AIaMDs) have received regulatory clearance internationally, with at least 343 devices cleared by the US Food and Drug Administration (FDA)^5^. In view of the rapid development of a large number of AlaMDs, the U.S. Food and Drug Administration (FDA), Health Canada, and the United Kingdom’s Medicines and Healthcare products Regulatory Agency (MHRA) have jointly identified 10 guiding principles that can inform the development of Good Machine Learning Practice (GMLP)^6^. These guiding principles will help promote safe, effective, and high-quality medical devices that use artificial intelligence and machine learning (AI/ML). There are major concerns regarding the presence of systemic, statistical and computational as well as human biases in AI^7^. In addition, there is major movement in the field of AI for the development of ethical AI^8^. This requires assessment of algorithms not only through the lens of performance but also through the various actors, processes, and objectives that drive the development and eventual deployment of the algorithm^8^.

Whole slide-based AI algorithms are often considered black boxes, as it is far from clear which features they are recognizing. The explainability is often restricted to a few features that by themselves would not explain the success of the algorithm. It has been argued that explainable AI will engender trust with the health-care workforce, provide transparency into the AI decision making process, and potentially mitigate various kinds of bias^9,10^. However, Ghassemi et al.^11^ suggest that this represents a false hope. They argue that rigorous internal and external validation of AI models could be a more direct means of achieving the goals often associated with explainability. They caution against having explainability be a requirement for clinically deployed models. In light of these comments, the work by Sandbank et al.^12^ provides a route to explainability by training algorithm on histological features. The CNN-based algorithm was developed to detect 51 different features associated with breast cancer. These features included cytological and morphological features of tumor cells in addition to other features such as inflammation, microcalcifications and adenosis.

Sandbank et al.^12^ have sought to develop and validate an assay for the detection of invasive and in situ breast carcinomas in a large series of cases. The initial work involved expert labor-intensive annotations and labeling of 1000s of areas from 2000 slides by a team of 18 pathologists. These 2000 slides were selected from a series of 115,000 slides to ensure representation of rare and unusual morphologies. Furthermore, to overcome the impact of domain shift, these cases were obtained from 9 different laboratories, each with their own pre-analytical variables. Initial training on a large number of cases with additional cross-laboratory training adds to the robustness of AI analysis. The failure to do so often leads to failure of many AI algorithms during the external validation.

The need for a large number of cases and associated manual annotation has been identified as a major bottleneck for AI analysis^13^. Newer methods are being developed that can circumvent these needs. Ren et al.^14^ have proposed that unsupervised domain adaptation could be performed using color normalization and/ or adversial training techniques. Unsupervised methods can be used to structure extremely large datasets. Similarly, self-supervised learning can be used to help models learn morphological, geometrical and contextual content of images using unlabeled data. Lastly, generative adversial networks (GAN) can be used to train on real images and synthesize realistic synthetic data; this can augment datasets and increase the performance of models with limited training^15^. Conditional GAN has been used for color normalization^16^. Janowczyk et al.^17^ have developed an open source quality control tool (HistoQC) for digital pathology slides to recognize and address the issues related to H&E quality.

Another important parameter for evaluation is the generalizability of the algorithm. Sandbank et al.^12^ validate their algorithm by obtaining slides/cases from two different institutions, stained with local methods (H&E and HES) which were scanned using 2 FDA-approved scanners. Furthermore, they use a large number of clinical cases to compare the diagnosis with expert pathologists. The analysis was performed on 5954 cases (12,031 slides) with alerts for invasive and in situ carcinoma. Invasive alert was raised for 363 (4.2%) of the slides of which 272 cases had been diagnosed as benign. Similarly, in situ alert was raised for 333 slides (3.8%; 237 cases). A review of these cases/ slides showed that 75% of the alerts were for necrosis, fibroadenomatous changes, hyperplasia or other features while 25% required additional workup to confirm or refute a malignant diagnosis; 2% of these called for additional second opinion. Overall the study showed that the algorithm could achieve a high AUC (0.99 for invasive cancer and 0.97 for in situ disease). This study design and output supports the notion that the algorithm could be generalizable.

The authors also took the opportunity to study concordance between the study pathologists and the original pathology report^12^. This analysis highlighted 11 discrepantly called cases between pathologists; seven had been called DCIS/ADH, while four cases were called benign. The review lead to the issuance of amended reports on these cases. From the patient management point of view, this indicates that the pathology labs misdiagnosed only four cases (an error rate of ~0.00067) for invasive cancer and 14 cases for in situ disease (an error rate of ~0.0023) out of 5954 cases, a remarkable performance.

The limitations of the study^12^ include the fact that the work was performed on biopsies and not excision specimens. The latter tend to be enriched for variants of benign lobules showing varying degrees of atrophy, in addition to other benign proliferations. However, the authors state that they were planning to extend the work in addressing these and other issues related to grading and assessment of margins. AI algorithms can be impacted by patient populations and healthcare disparities. Furthermore, they can systematically mis-represent and exacerbate health problems in minority populations^18,19^. Although the racial distribution of the patient population is not provided, the current study was involved assessment of the algorithm in a large metropolitan area, which is likely to have multi-ethnic patient population. Furthermore, it is unlikely that the patient ethnicity and health inequities will affect the performance of an algorithm developed for the histological diagnosis of cancer.

Overall, this work offers an excellent blue print for the development and validation of algorithms in digital pathology. The main question before us now is what degree of validation is necessary prior to clinical deployment of the algorithm as a second-read system. Is the development and validation in 7485 cases (15,124 slides) from at least nine different institutions sufficient? Is an error rate of a few percentage points good enough? I for one, would gladly accept such a tool to prevent the less than 0.001% error that pathologists make. The question, however, ultimately boils down to the cost of doing the second reads and what the patients and payers are ready to accept as human error.
